# Wood-Based Panels and Volatile Organic Compounds (VOCs): An Overview on Production, Emission Sources and Analysis

**DOI:** 10.3390/molecules30153195

**Published:** 2025-07-30

**Authors:** Fátima Daniela Gonçalves, Luísa Hora Carvalho, José António Rodrigues, Rui Miguel Ramos

**Affiliations:** 1 LAQV-REQUIMTE—Departamento de Química e Bioquímica, Faculdade de Ciências, Universidade do Porto, Rua do Campo Alegre, s/n, 4169-007 Porto, Portugal; up201805253@up.pt (F.D.G.); jarodrig@fc.up.pt (J.A.R.); 2 DEMad—Departamento de Engenharia de Madeiras, Instituto Politécnico de Viseu, Campus Politécnico de Repeses, 3504-510 Viseu, Portugal; lhcarvalho@estgv.ipv.pt; 3 LEPABE—Faculty of Engineering, University of Porto, Rua Dr. Roberto Frias, s/n, 4200-465 Porto, Portugal; 4 ALiCE—Associate Laboratory in Chemical Engineering, Faculty of Engineering, University of Porto, Rua Dr. Roberto Frias, 4200-465 Porto, Portugal; 5 ARCP CoLAB—Rede de Competência em Polímeros, UPTEC Asprela II, Rua Júlio de Matos 828/882, 4200-355 Porto, Portugal

**Keywords:** emission, indoor air quality, volatile organic compounds, wood, wood-based panels

## Abstract

The emission and presence of volatile organic compounds (VOCs) in the indoor air of houses and factories has been a growing topic of debate in the industry and related research fields. Given the extended times people in modern society spend indoors, monitoring VOCs is crucial due to the associated potential health hazards, with formaldehyde being particularly noteworthy. Wood and wood-based panels (WBPs) (the latter constituting a significant segment of the wood-transforming industry, being widely used in furniture, construction, and other applications) are known sources for the emission of VOCs to indoor air. In the case of the WBPs, the emission of VOCs depends on the type and species of wood, together with industrial processing and addition of additives. This review integrates perspectives on the production processes associated with WBPs, together with the evolving global regulations, and thoroughly examines VOC sources associated with WBPs, health risks from exposure, and current analytical methods utilized for VOC detection. It comprises an overview of the WBP industry, providing relevant definitions, descriptions of manufacturing processes and adhesive use, analysis of legal constraints, and explanations of VOC source identification and describing analysis techniques utilized for VOCs in WBPs.

## 1. Contextualization

Volatile organic compounds (VOCs) are known air pollutants emitted from a variety of indoor and outdoor sources. Considering that the lifestyles associated with modern society require that significant periods of time be spent in indoor environments, in turn leading to extended periods of exposure, it is important to be aware and actively work toward a more careful monitoring of these compounds. Wood-based panels (WBPs) have been a fast-growing branch of the wood-transforming industry and have found a place in homes worldwide, in furniture, cabinets, doors, flooring, and other construction materials. They have been actively linked to the emission of several potentially toxic VOCs, with the most relevant being formaldehyde. While formaldehyde, due to its classification as a carcinogenic compound (Group 1) by the International Agency for Research on Cancer (IARC), is a predominant topic in WBPs and general indoor-air quality publications [1,2,3,4,5], the same focus is not given to the broader spectrum of VOCs that can be released from these products. This work offers a multidisciplinary view of WBPs, from production to legislation, describing their role as a source of VOCs and the different emitting classes associated with WBPs, as well as the health hazards resulting from VOC exposure and the analytical methods currently available to be utilized for their control.

This review will be structured into five major sections:

1. Contextualization: Presents the objectives and structure of the work;
2. Wood-based panels: Contains an overview of the wood and WBP industry, the different WBPs and their manufacturing processes, the adhesives employed, and the legal restrictions the production of WBPs must abide by;
3. Volatile Organic Compounds Emitted from WBPs: Focuses on VOCs, specifically, the identification of possible sources within WBPs, followed by an in-depth discussion of the most prominent groups of compounds emitted;
4. Methodologies Used for the Analysis of VOCs in WBPs: Presents both the standard methods used for the determination of VOCs in WBPs and alternative analytical methods;
5. Comparison of Emission Results Found in the Literature: Provides a summary and discussion of the determined values for terpenes, carbonyl compounds, and others for different types of wood and WBPs.

## 2. Wood-Based Panels

### 2.1. Wood

Wood, known for its availability and unique properties, is considered the most abundant renewable and biodegradable resource [6]. As one of the oldest materials used by humans for energy generation and construction purposes, the overexploitation of this natural resource has become environmentally and economically unsustainable. Forests are natural ecosystems that not only provide raw materials and various non-wood products but also play important roles in carbon storage, biodiversity preservation, and climate regulation [7]. Therefore, intense harvesting leads to concerning consequences, such as diminishing the ability of these ecosystems to capture the ever-abundant atmospheric CO_2_, one of the main greenhouse gases [8,9]. Decreasing availability and increasing costs of wood logs and lumber over the years have highlighted the need to develop sustainable ways to use this resource more efficiently [10].

One sector of the wood industry that can use wood waste as a secondary material source is the WBPs industry. According to the Wood4Bauhaus, more than 75% of the wood fibre used by the European Panel Federation (EPF) is either recovered wood or is from by-products of the wood and WBP industries [11].

### 2.2. Production of Wood-Based Panels

WBPs encompass a diverse range of board products manufactured from various forms of wood, such as fibers, particles, strands, or veneers, glued together by a thermosetting adhesive and consolidated through the application of heat and pressure [10,12]. Depending on their intended use, these WBPs can be classified into structural panels and non-structural panels and can be used for dry, humid, or external conditions [13,14,15]. According to EN 13986:2004+A1:2015, WBP can refer to solid wood panels, laminated veneer lumber, plywood, oriented strand board (OSB), resin-bonded particleboard (PB), cement-bonded particleboard, or fiberboard.

The production of a WBP begins with the preparation of the wood fibers or particles. When wood is obtained from a primary source (e.g., virgin lumber), the wood logs must be debarked before being broken down into smaller pieces or cut into sheets, as the bark can compromise the quality and performance of a panel [16,17]. This processing is typically carried out using machinery equipped with blades, hammers, or grinding disks to achieve the different sizes of wood elements (chips, flakes, particles, fibers, etc.) needed for the different panels. Following the preparation of the wood elements, they are blended with adhesive and other additives (e.g., catalyst) during the blending stage. The initial shape of a panel is first obtained in the mattress forming stage, which can be followed by a pre-pressing step and then the hot-pressing stage. Post-production operations such as trimming the edges, cutting to size and sanding are performed after cooling [10,18].

#### 2.2.1. Plywood

Plywood, as defined in EN 313-2:2010 [19], consists of an assembly of layers (veneers) glued together with the direction of the grain in adjacent layers usually at right angles [20]. Given the nature of its wood element, the veneers must be obtained directly from a primary source (lumber) [10]. The logs are first conditioned to appropriate temperatures to prevent sheet damage caused by shattering and then positioned in a peeler to be cut into veneers. Following the veneer processing stage, the veneers are dried prior to adhesive spreading. A loose mattress is formed, typically by alternating the grain direction of the veneers. This is followed by the pre-pressing and hot-pressing stages [6,16].

Plywood panels can be used for various purposes. According to the EPF, 38% of the plywood produced in 2022 was used in construction (roofing, sheathing, and flooring), 27% in furniture manufacture (tables, chairs, and cabinets), 8% in packaging (shipping crates, and boxes), and 14% in other uses, including decoration (interior paneling) [21,22]. Plywood may also be found in the automotive (structured interior building, molds, and templates) and boat building industries (structured interior building, hulls, and decks), which together accounted for 13% of its usage. Despite its wide range of applications, plywood has been progressively replaced by OSB due to its higher cost [22,23].

#### 2.2.2. Oriented Strand Board

Similar to plywood, OSB, as defined in EN 300:2006 [24], is a multilayered panel produced from thin strands of raw-wood (flakes) bound together with a binder [10,16]. The wood processing steps for OSB are very similar to those used for plywood panels, while the manufacturing of the panel is closer to that of particleboard [17]. As only virgin wood is used in its manufacturing, the mechanical performance of these panels is comparable to that of structural plywood [22]. 

The conditioned wood is fed into a ring flaker to slice the wood into flakes. The wood strands that make up the outer layers are usually long and positioned along the same orientation of the board, while the strands present in the core layers are smaller and may be oriented perpendicular to one another or randomly positioned [16,17]. This perpendicular alignment of long strands in adjacent layers is characteristic of OSB panels and is reflected in the strength of the finished product. Pre-pressing is not necessary in OSB manufacture.

The most frequent application of OSB panels is for construction purposes, which correspond to 80% of the end-uses seen by the EPF in 2022, followed by packaging (6%), flooring (5%), furniture (3%), and Do-It-Yourself (2%) [25].

#### 2.2.3. Particleboard

As defined in EN 309:2005, the wood (virgin or recycled) used in PB production is processed into particles, dried in a slow-rotating dryer to remove most of its moisture content, and then sorted according to size by a sieve machine to select those with desirable dimensions [18,26]. Of these particles, the smaller ones, which are similar in size and shape, are used in the faces of the board, while the core of a PB comprises the bulkier and more irregular particles [10,14]. 

The adhesive and other additives such as wax, hardeners, and preservatives are added and slowly mixed with the particles in a blender. The mixture resulting from the blending process is then shaped into a panel by a mattress former, where the face and core adhesive-particle blends are added in a face-core-face order on a continuous conveyor by spreader heads [10]. A cold pre-pressing step usually follows to remove trapped air inside the mattress. 

**Figure 1:**
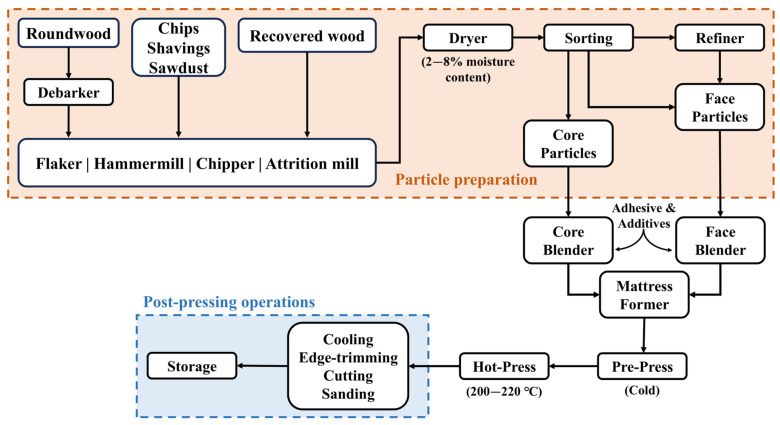
Schematic diagram of the particleboard production process. Adapted from [10,18].

The final production stage is the hot-pressing stage, in which the heat activates the adhesive, leading it to harden, while the pressure of the press aids in binding the particles together [17]. The production process of a PB is summarized in Figure 1.

As of 2022, 66% of the PBs produced in the European Union were used in the manufacture of furniture, usually with a veneered or laminated finish. PBs used in construction—including flooring and door applications—corresponded to 27%, while 1% went towards packaging [27].

#### 2.2.4. Medium-Density Fiberboard

Officially designated as dry-process fiberboard [20], the production of medium-density fiberboard (MDF) panels shares similarities with the production of PB. However, there are major differences in the fiber preparation and blending steps [18]. 

**Figure 2:**
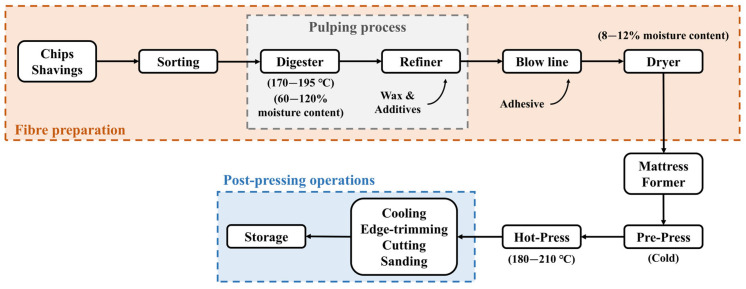
Schematic diagram of the medium-density fiberboard production process. Adapted from [10,18].

Wood fibers are usually produced through a thermomechanical pulping process in a refiner, which uses high temperatures and humidity to turn chips and shavings into soft fiber bundles [10]. These bundles are then blended with the adhesive and other additives added as aqueous solutions and dried to obtain resin-coated fibers with a moisture content lower than 20% (dry-process) [10,17]. The remaining production stages are carried out similarly to those used in PB production. The production process of MDF is summarized in Figure 2.

MDFs are stable, homogenous panels with smooth surfaces, which makes them suitable for the application of decorative finishes or for painting. As of 2022, the EPF reported the use of MDFs in furniture manufacturing (51%), in laminated flooring (19%), in construction (14%), and in packaging and molding (10%) [27].

### 2.3. Adhesives in WBP Production

The adhesives used in WBP production can be classified as natural or synthetic, and further categorized into thermosetting and thermoplastic resins. Among synthetic adhesives, the focus is on thermosetting resins, particularly amino resins (such as urea-formaldehyde (UF) and melamine-urea-formaldehyde (MUF)), phenol-formaldehyde resins (PF), and isocyanate-based resins (pMDI), which are commonly used in wood composite production [28,29].

While UF resins are widely used due to their low cost, adaptability to curing conditions, and fast curing, there are also known limitations to their use. They are more susceptible to degradation under humid or acidic conditions and slowly release formaldehyde from the finished panel [28,29,30,31]. To mitigate formaldehyde emissions, adjusting the formaldehyde/urea ratio during resin synthesis is a common practice.

MUF resins, when compared to UF resins, exhibit higher moisture resistance, which makes them more appropriate for humid conditions in interior applications or covered outdoor applications. Although the incorporation of up to 40% of melamine in the formation of MUF resin increases the moisture resistance of the wood products in which it is used, it also results in higher costs, as melamine is less affordable than urea [28].

PF resins are highly stable adhesives, displaying significant resistance to hydrolysis degradation and minimal formaldehyde emissions, particularly in comparison to UF resins [28,29,30,31]. These resins find extensive use in the manufacture of exterior panels like construction plywood and oriented-strand board. However, their higher cost and the tendency to yield panels with reduced mechanical properties remain noteworthy drawbacks.

Isocyanate-based resins represent a more costly but highly durable option for exterior-grade adhesives, predominantly utilized in the production of OSB. The absence of formaldehyde and other VOC emissions upon curing is a major advantage of this adhesive system [31]. However, challenges arise due to pMDI’s strong adhesion to the press plates or belts, necessitating the use of appropriate release agents, as well as its associated handling risks [32].

A summarized comparison of these different synthetic thermosetting resin systems listing their applications, advantages, and disadvantages, is presented in Table 1.

Bio-based resins are natural adhesives that should exhibit similar performance to synthetic resins while being derived from natural and organic sources. These resins can be based on proteins, tannins, carbohydrates, oils, and liquefied cellulosic materials [31]. Although interest in these adhesives has increased in recent years, their impact on the market is still far from surpassing that of synthetic resins.

### 2.4. Emission Issues and Legislation

Panels produced with formaldehyde-based adhesives are prone to release formaldehyde, which is a serious concern as the main applications of WBP are the construction and furniture sectors. In an effort to regulate the impact of these emissions, several countries impose emission limits on different types of panels.

According to the European Standard EN 13986:2004+A1:2015 [15], panels with formaldehyde-containing materials as part of their production process must be tested and classified into the E1 or E2 categories, according to their formaldehyde content or emission. The formaldehyde content for panel classification is determined by the perforator method (ISO 12460-5) [33], while formaldehyde emission is determined by the chamber method (EN 717-1) [34]. To be classified as an E1 product, the formaldehyde content in the panel must be less than or equal to 8 mg formaldehyde/100 g of dry board, and formaldehyde emission must not exceed 0.124 mg·m^−3^ air. A panel with a formaldehyde content higher than 8 mg but not greater than 30 mg per 100 g of dry board or, formaldehyde emissions higher than 0.124 mg·m^−3^ air, is classified as an E2 product. 

In 2023, the European Commission established a new class E0.5 with a maximum emission limit for formaldehyde in wooden articles and furniture at 0.062 mg·m^−3^ air (half of the emission limit of class E1), which will be introduced in a revised version of EN 13986:2004+A1:2015 and will become compulsory from July 2026 [35].

The Environmental Protection Agency of the United States of America (USA) issued limitations on formaldehyde emissions in different WBPs in accordance with the California Air Resources Board, based on a large chamber method ASTM E 1333 [36]. After 2018, formaldehyde emissions in WBP produced and/or circulating in the USA must not exceed 0.05 ppm (0.061 mg·m^−3^ air) for hardwood plywood panels produced with a veneer or composite core, 0.11 ppm (0.135 mg·m^−3^ air) for MDF, 0.13 ppm (0.160 mg·m^−3^ air) for thin MDF and 0.09 ppm (0.111 mg·m^−3^ air) for PB [37].

In 2021, China updated its regulation for the classification of indoor wood-based panels and their products in accordance with its formaldehyde emissions in GB/T 39600-2021 [38]. The Chinese E1 corresponds to the European E1 (0.124 mg·m^−3^ air), while the Chinese E0 refers to panels with formaldehyde emissions higher than 0.025 mg·m^−3^ air but not exceeding 0.050 mg·m^−3^ air. Grade ENF is used to classify panels with formaldehyde emissions equal to or lower than 0.025 mg·m^−3^ air [39].

In Japan, the Japanese industrial standard JIS A 5908 [40] classifies particleboards according to their formaldehyde emissions by the desiccator method, described in JIS A 1460 [41], into F**** (≤0.3 mg·L^−1^), F*** (≤0.5 mg·L^−1^) and F** (≤1.5 mg·L^−1^).

While formaldehyde is a compound with mandatory regulation in panel production, it is not the only harmful VOC emitted from WBPs. Other VOCs are also emitted, although only a few countries have set legal limits concerning VOC emissions from construction products. The legal limits on Total VOC (TVOCs) and specific VOC emissions from construction products in different countries are summarized in Table 2 and Table 3.

## 3. Volatile Organic Compounds Emitted from WBPs

Wood, WBPs, and their products are present in most indoor environments, representing an important indoor source of VOCs. Several factors can influence the emission of VOCs from these products, such as the nature of the wood material and the degradation of its components, the addition of resins, varnishes, or other additives, and the steps involved during their industrial production. The composition of wood can be divided into the main structural components (cellulose, hemicellulose and lignin) which are common in all types of wood, and the minor low-molecular-weight non-structural components (usually up to 10%) which are characteristic of a wood species in type and quantity [45,46]. VOCs such as terpenes, terpenoids, alcohols, aldehydes, and ketones constitute an important portion of wood extractives [47,48].

The type and amount of VOCs present and emitted from natural wood, and therefore, the wood material of WBPs, are significantly dependent on wood type and species [48], as well as factors such as age [49,50], weather and environmental conditions [47], time of cutting, storage conditions and drying conditions [50,51]. Furthermore, although significant differences have been reported in the content of VOCs emitted from the sapwood and heartwood regions, the overall composition of the emitted compounds is very similar [52,53]. 

Softwood is used to designate wood from conifer trees (e.g., pine, spruce, larch, fir), which are rich in resin acids, terpenes, fatty acids, and flavonoids [54]. Although volatile terpenes can make up to 90% of softwood VOC emissions, they also emit low amounts of secondary emissions (aldehydes and acids) [48]. Flowering trees or angiosperms such as birch, cherry, maple, and oak are designated as hardwoods. In contrast to softwoods, hardwoods have significantly lower emissions of terpenes, as volatile terpenes are usually absent; however, these woods emit a variety of secondary emissions such as aldehydes, ketones, carboxylic acids, and alcohols [48]. 

An in-depth overview of VOCs found in wood and WBPs, together with their wood source, the analytical method used for their determination and the content found is available in the Supporting Information.

### 3.1. Terpenes and Terpenoids

Terpenes are hydrocarbon compounds and the largest class of plant secondary metabolites, usually serving as defensive molecules against biogenic enemies or as signal compounds to e.g., attract pollinators [55,56,57]. Terpenes can undergo several reactions such as hydrogenation, dehydrogenation, and oxygenation to form terpenoids [58,59]; however, the term “terpene” can also be used to include terpenoids.

Terpenes are composed of isoprene units (2-methyl-1,3-butadiene) and can be classified according to the number of isoprene units or carbon atoms. The number of isoprene units is related to a terpene’s volatility, as fewer isoprene units are associated with higher volatility. Volatile terpenes are part of wood’s primary VOC emissions and include hemiterpenes (1 unit, C5), monoterpenes (2 units, C10), and sesquiterpenes (3 units, C15) [60].

The presence of terpenes in wood is primarily linked to natural resin, as the sapwood cells through which resin flows contain volatile and non-volatile terpenes in their composition [54], and are largely responsible for the characteristic fragrant smell of wood [45,48]. Due to being rich in natural resin, softwoods not only have characteristically high emissions of monoterpenes and monoterpenoids, but also emit several sesquiterpenes and sesquiterpenoids [59]. 

Of the many softwood species studied over the years, the highest concentrations of VOCs have been found in the pine family species, where terpene emissions predominate significantly in their VOC profile. Compounds such as α-pinene, Δ3-carene, β-pinene, and limonene are characteristic volatile terpenes of coniferous wood species; moreover, camphene, myrcene, and β-phellandrene are also frequently found in softwoods, although at lower amounts. A more extensive list of the volatile terpenes emitted from pine, spruce, larch, and fir can be seen in Table 4.

Prolonged storage or exposure to high temperatures—such as those used for drying and thermal treatment or hot-pressing—can significantly reduce terpene emissions in wood and WBPs. High temperatures are known to cause most volatiles to escape the wood material or to degrade into other compounds during heat treatment, which is evidenced by the high terpene emission rates obtained from studies conducted in a chip dryer [71]. 

In a comparative study of untreated and heat-treated pine (*Pinus sylvestris* and *Pinus* spp.), emissions of the most abundant terpenes, α-pinene and limonene, decreased by 85–89% and 95%, respectively. Additionally, compounds like camphene, β-phellandrene, and β-myrcene were no longer emitted by the heat-treated pine samples [65,66]. Similar findings were observed in spruce (*Picea abies*), where emissions of α-pinene and Δ3-carene decreased by 99%, and limonene by 79%. Heat-treated spruce also showed no emissions of β-phellandrene or β-myrcene [66].

This same behavior is reflected in WBPs produced with softwoods. In a study performed by Que et al. (2013) [72], an assessment of VOC emissions from several WBPs showed that VOC emissions from fresh PB (2 weeks old) were four times higher than those from a 10-year-old PB, in which α-pinene and Δ3-carene were the most noticeable terpenes. Emissions from non-resinated wood sawdust and shavings, obtained from the same source as the 2-weeks-old PB, had a similar terpene composition. 

In 2013, Son et al. [73] determined the emission factors of natural VOCs in different softwood lumber and WBPs such as PB, MDF and plywood. A significant decrease in emissions was seen after 10 days of analysis and when comparing the emission factors resulted from the several lumber samples to those from WBPs. These PB and MDFs, which were produced with waste wood and UF resin, had α-pinene, β-pinene and Δ3-carene as their highest emitted compounds, as well as lower emissions of limonene, β-myrcene, camphene, α-terpinene, and γ-terpinene. 

In a study from Baumann et al. (1999) [74], 57 PB and MDF samples produced with southern pine, other pine, Douglas fir (*Pseudotsuga menziesii*) (PB only) and hardwood were analyzed to determine their terpene composition [74]. α-pinene and β-pinene were quantified in all PB samples, showing higher results for both pine groups. PB produced from hardwood only showed emissions of α-pinene, β-pinene and Δ3-carene, which were significantly lower than in any other PB samples. Furthermore, MDF samples were mostly absent of terpene emissions. 

As mentioned before, hardwoods have significantly lower VOC emissions than softwoods as they mainly contain non-volatile higher terpenes [45,59] and only a few tropical hardwoods have been reported to emit monoterpenes, such as camphor from *Cinnamomum camphora* [45]. A list of volatile terpenes reported in particleboards and MDFs produced with different wood types can be seen in Table 5.

Nowadays, with the increasing number of possible emission sources, the possibility of continuous VOCs exposure represents an important health concern. Exposure to VOCs and their consequences to human health has been increasingly documented, with reports targeting each class of compounds discussed in this insight.

Terpenes are mostly associated with symptoms of irritation affecting the eyes, mouth, respiratory system, and skin [78]. According to Falk et al. (1991) [79], Δ3-carene may cause irritation of the skin and mucous membranes while prolonged exposure may lead to allergic contact dermatitis or chronic impairment of lung function.

### 3.2. Aldehydes, Carboxylic Acids, and Ketones

Another important group of VOC emitted from wood and WBP are the carbonyl compounds, which include aldehydes, carboxylic acids, and ketones.

Most aldehydes derive from the oxidative degradation [2,48,50,59,80] and/or thermal degradation [2,59] of components abundant in wood. These processes may occur naturally or be a consequence of WBP manufacturing. Biogenic formaldehyde is present in low concentrations and may occur through the oxidation of unsaturated terpenes such as α-pinene, β-pinene, and limonene, as well as due to the decomposition of lignin [2,50,59]. In contrast, anthropogenic formaldehyde results from the synthetic resin added in WBP production [50,81]. Authors Risholm-Sundman et al. [48] and Roffael [80] suggest that oxidation of unsaturated fatty acids in wood leads to the formation of saturated aliphatic aldehydes, of which hexanal is usually the most abundant. Furthermore, pyrolysis of wood leads to the breakdown of polysaccharides and results in compounds such as formaldehyde, acetaldehyde, propenal, and butanone. Thermal degradation of lignin produces benzaldehyde and other aromatic compounds [2,59].

Formic, acetic, and hexanoic acids are organic acids widely emitted in relatively low amounts from wood, although other acids may appear in specific wood types [59]. Acetic acid may occur due to the hydrolysis and cleavage of acetyl groups in lignin and hemicellulose [48]. Formic and hexanoic acids may originate from the decay of fatty acids (and further degradation of formaldehyde and hexanal, respectively) [82].

The formation of organic acids and/or exposure to high temperatures leads to the degradation of polysaccharides, resulting in the formation of furan aldehydes and derivatives such as furfural (furan-2-carbaldehyde), 5-hydroxymethylfurfural and 5-methylfurfural [45,83,84,85,86]. Their formation may also occur through the hydrolysis of carbohydrates [51].

Acetone is the most emitted ketone from wood and WBP. However, emissions of other ketones such as acetophenone [64], 6-methyl-5-hepten-2-one, and hydroxypropan-2-one (hydroxyacetone/acetol) [87] have also been reported. While a study showed a possible correlation between acetone emissions from pine trees and photosynthesis [88] and another reported the formation of acetone by the photolytic degradation of cellulose [89], little is known about the mechanism behind the formation and emission of acetone in wood. Although some mechanisms responsible for the formation of certain aldehydes and ketones through thermal, enzymatic, and microbial degradation have been studied, they fail to explain the diverse range of compounds detected. Furthermore, these mechanisms may occur in conditions significantly different from those encountered in the WBP manufacturing [2].

Although hardwoods have substantially lower emissions than softwoods due to their general lack of volatile terpenes, they have considerably high carbonyl emissions (Table 6) [45,48]. Hardwood hemicelluloses have a higher number of acetyl groups. Therefore, the concentration of acetic acid emitted from hardwood is usually higher than that from softwoods [45]. 

A study from Risholm-Sundman et al. (1998) [48] focused on the emissions of acetic acid and other VOCs from several wood species. Results showed significantly higher acetic acid emissions for oak (*Quercus robur*) and cherry (*Prunus serotina*), followed by rubberwood (*Hevea brasiliensis*), ash (*Franxinus excelsior*), maple (*Acer saccharum*), and beech (*Fagus sylvatica*). Birch wood (*Betula pubescens*) was the only hardwood sample to show lower acetic acid emissions than the two softwoods tested (pine and spruce). Regarding other carbonyl compounds, hexanal was among the most emitted compounds from all wood samples excluding ash and rubberwood. Beech, birch, and pine showed considerable acetone emissions.

Wood drying and pressing procedures in the WBP manufacturing process also affect the emissions of carbonyl compounds, although this effect differs from observed with terpenes. 

Manninen et al. (2002) [87] compared air-dried and heat-treated pine wood (*Pinus sylvestris*) and found that terpene emissions were higher in air-dried samples, which exhibited a lower variety and percentage of aldehydes. In contrast, heat-treated samples showed significant differences in their emission profiles. The major compounds emitted in heat-treated pine were acetic acid (23.91%), furfural (28.37%), and acetone (7.34%), which were absent from air-dried pine samples as they occur through thermal degradation. Other carbonyl compounds identified were hydroxyacetone, hexanal, octanal, nonanal, decanal, benzaldehyde, and 5-methylfurfural. Overall, aldehydes contributed 34.54% to the total VOC emissions from heat-treated wood, followed by carboxylic acids and their esters (30.64%) and ketones (16.20%). Terpene emissions accounted for only 9.69%, contrasting to the 77.03% emitted from air-dried pine.

Similarly, a study from Wang et al. (2018) [65], which compared untreated pine and heat-treated southern yellow pine (*Pinus* spp.), concluded that a higher percentage of carbonyl emissions occurred in heat-treated samples. In 2010, benzoic acid was also reported in heat-treated pine (*Pinus sylvestris*), while the hardwood aspen (*Populus tremula*) emitted benzoic, propanoic and hexanoic acids from both air-dried and heat-treated samples. Furthermore, the emission rates of organic acids and aldehydes were shown to remain constant throughout 4 weeks, regardless of wood type (softwood or hardwood) or drying treatment (air-dried or heat treatment) [66].

Baumann et al. (2000) [94] studied aldehyde emissions from PBs and MDFs produced with wood from different species. The study identified pentanal, hexanal, heptanal, benzaldehyde, octanal, *trans*-oct-2-enal, and nonanal as the most predominant aldehydes in both products. Formaldehyde was not detected by the GC-MS method used, due to being too volatile. Overall, MDF emissions were substantially lower than PB emissions, except for panels made from southern pine. Hexanal was the most significant contributor to total VOC emissions across most samples, namely Douglas fir PBs (41%), other pine PBs (44%), southern pine PBs (48%), other pine MDFs (47%), southern pine MDFs (58%), other PBs with undetermined wood (68%) and hardwood PBs (80%). Aldehyde emissions were higher than non-aldehyde emissions in all panels, except for Douglas fir PBs (49%), hardwood MDFs (40%), and other MDFs with undetermined wood (16%).

In a study from Liu et al. (2020) [77], emissions from PB and laminated PB—both produced with the same wood particles—were analyzed and several volatile groups were identified in both samples. Among them were carbonyl compounds such as acetaldehyde and straight chained aldehydes ranging from C_6_ to C_11_, methylal (dimethoxymethane), acetone, ethyl acetate, methyl methacrylate, and acetic acid esters.

The use of additives, such as flame retardants, has also been reported to influence the VOCs emitted from wood and WBPs. In 2023, Fuczek et al. [95] studied the effect of ammonium sulphate, an acid-based flame retardant, on lightweight insulation fiberboard. Results showed a noticeable increase in the emissions of acetic acid and furfural when compared to panels without the additive. 

More recently, Trojanová et al. (2025) [96] compared emissions from untreated and flame retardant-treated spruce wood (*Picea abies*) under thermal loading. The findings indicated that furfural was the main emission in flame retardant-treated spruce.

A list of volatile carbonyl compounds emitted from PBs and MDFs produced with different wood types can be seen in Table 7.

General health risks associated with aldehyde exposure include symptoms such as irritation of the eyes, nose, and throat, as well as headaches at lower concentrations. At higher concentrations, exposure may lead to respiratory damage, illnesses, and even cancer. 

According to the International Agency for Research on Cancer (IARC), and later adopted in the regulation of Registration, Evaluation, Authorisation and Restriction of Chemicals (REACH) by the European Chemicals Agency (ECHA), formaldehyde is classified as a Group 1 carcinogen. It is known to cause nasopharyngeal cancer, suspected to be associated with leukemia [99]. Inhalation of low doses of formaldehyde can result in symptoms such as headaches, shortness of breath, and rhinitis. Exposure to higher concentrations may cause excessive tearing [100], irritation of the mucous membrane, and a range of respiratory issues, including asthma, bronchitis, pulmonary oedema, and pneumonia [101,102]. 

Acetaldehyde is currently categorized by IARC as a possible human carcinogen (Group 2B) [103]. Acute exposure to significant levels of acetaldehyde have been reported to cause irritation of eyes, nose, and throat [104].

Although present in significantly lower concentrations than terpenes and aldehydes, acetic acid is also noteworthy of concern. Both short-term and long-term exposure, especially in individuals with seasonal allergies, may induce asthma-like symptoms [105].

### 3.3. Other Compounds

Emissions of alcohols have been reported in different wood species; however, no clear formation mechanism was found in the literature. Methanol is among the most prominent alcohols emitted from several heat-treated hardwoods [48], while ethanol [65,87], 1-octanol, and 2-methoxyphenol have been found in heat-treated pine [65]. Comparisons between air-dried and heat-treated emissions from softwood (pine and spruce) and hardwood (aspen), compounds such as 1-hexanol, 1-pentanol and 1-penten-3-ol were found in air-dried aspen while heat-treated aspen samples resulted in low emissions of 1-butanol. 1-pentanol was also present in emissions from air-dried pine [66]. Fresh wood emissions have also included alcohols such as pentan-1-ol found in pine, pronan-2-ol in larch, and 2-ethylhexanol in both larch and spruce [64].

The thermal decomposition of lignin may also result in the formation of aliphatic and aromatic hydrocarbons, phenol, and phenolic compounds. McGraw et al. (1999) reported that some terpenes, including Δ3-carene, limonene, and camphene, can degrade into volatile aromatic compounds such as *p*-cymenene and *o*-cymene [106]. After thermal modification, vanillin and guaiacylacetone were identified in three Tunisian pine species [107], whereas anetol (or estragole) was found to be the most abundant aromatic compound emitted from southern yellow pine (*Pinus* spp.) [65]. Other identified aromatic hydrocarbons in PB and laminated PB include C_10_–C_16_ alkanes, benzene, toluene, naphthalene and their derivatives, *p*-xylene, and phenanthrene [77]. Furans and its derivatives—such as alkylfurans (ethyl-, propyl- and pentyl-)—were detected in hardwoods as products of thermal degradation of cellulose and other polysaccharides [108,109]. Additionally, thermal degradation of hemicellulose may lead to the formation of esters, which occur through reactions between hydroxyl and carboxyl groups at high temperatures [59]. A summary of other volatile compounds found in different wood types is presented in Table 8.

## 4. Methodologies Used for the Analysis of VOCs in WBPs

### 4.1. International Standards

The ISO 16000 series addresses the assessment of indoor air quality and is divided into several parts. Among these, a select number specify standardized methodologies for the determination of VOCs in indoor air.

The active sampling method described in ISO 16000-3 [110] enables the determination of formaldehyde and 12 other volatile carbonyl compounds in the air inside a test chamber and in indoor air. The air is drawn through a silica gel cartridge coated with 2,4-dinitrophenylhydrazine (DNPH) reagent, where the carbonyl compounds will form stable derivatives with DNPH. The resulting derivatives are analyzed by high-performance liquid chromatography (HPLC) with ultraviolet detection or diode array detection (DAD). This method can be used for short-term (5 min to an hour) and long-term (1 h to 24 h) air sampling, and the results are usually expressed in parts per million, ppm.

In ISO 16000-6 [111], the determination of VOCs emitted from building materials and other products used in indoor environments is specified. Sampled air from an emission test chamber or cell is drawn through one or more Tenax TA^®^ sorbent tubes to capture the analytes. By means of thermal desorption, the captured VOCs are analyzed by gas chromatography (GC) using a flame ionization detector and/or a mass spectrometry (MS) detector. The concentration of the identified individual VOCs is expressed in µg·m^−3^.

ISO 16000-9 [112] specifies a method for determining the area-specific emission rate of VOCs from building products and furnishings using an emission test chamber. To determine the area-specific emission rate by the concentration of VOCs in the air, the test must be conducted in an emission test chamber at a constant temperature of 23 °C, relative humidity of 50% and area-specific air flow rate. Air sampling shall occur at 72 h and 28 days after the start of the test. The air sampling process and the analytical method for the determination of VOCs are the same as described in ISO 16000-6. The concentration of individual compounds and/or total VOCs is expressed in µg·m^−3^.

Regarding emissions of regulated hazardous substances from construction products into indoor air, the EN 16516 [42] describes a horizontal reference method based on the test chamber which allows the determination of VOCs, very volatile carbonyl compounds and ammonia. Chamber conditions are the same as those described in ISO 16000-9. The air sampling and analysis of VOCs is performed in a similar manner to that described in ISO 16000-6, while the determination of formaldehyde, acetaldehyde, propanal, butanal and acetone occurs similarly to ISO 16000-3.

Although these methods use a test chamber, which allows an accurate representation of an indoor environment during sampling, they are also associated with high costs and often long sampling times. For example, ISO 16000-9 defines a 28-day sampling period. This extended duration can pose a challenge for routine internal emissions monitoring in the WBP industry.

### 4.2. Other Methods

As an alternative to the standardized methodologies described above, several other techniques allow the extraction of VOCs either directly from wood and WBP samples or by sampling the volatiles they emit.

Classic extraction methods rely on solid-liquid extraction, aiming to isolate compounds from a solid matrix through a liquid solvent. The efficiency of extraction depends primarily on the solvent’s polarity and the chemical nature of the target compounds. Therefore, solvent selection is a critical step. Commonly used solvents include methanol, ethanol, acetone, dichloromethane, and n-hexane, which are often applied as mixtures or with varying percentages of water to improve yield [54]. Other variables, such as the temperature, pressure, extraction time, and the characteristics of the solid sample also influence the outcome of the extraction [113]. Among the classic extraction procedures most frequently used are Soxhlet, hydrodistillation, and steam distillation.

In 2007, Vichi et al. [114] employed the use of accelerated solvent extraction (ASE) coupled with GC-MS to extract and determine volatile and semi-volatile compounds in oak wood chips, having detected the presence of over 90 compounds and identified most of these. Similarly, Bukhanko et al. (2020) [115] determined several natural volatile compounds from Norway spruce, such as terpenes and aromatic compounds, in extracts obtained by Soxhlet extraction and analyzed by GC-MS. Bertaud et al. (2017) [116] reported the extraction of volatile terpenes from spruce (*Picea abies*), fir (*Abies alba*), and maritime pine (*Pinus pinaster*) by different extraction methods, namely Soxhlet, ASE, steam distillation and supercritical CO_2_ extraction. For the conditions used, Soxhlet extraction resulted in a higher concentration of terpenes.

A widely used alternative technique for capturing VOCs emitted directly from solid samples is solid-phase microextraction (SPME). Originally developed by Pawliszyn [117], SPME is a sensitive, solvent-free, and non-exhaustive technique based on establishing equilibrium between the sample and the extraction phases. It has been applied to analyze volatile and semi-volatile compounds from various sample matrices, such as wines [118,119], wood [53,69], soil [120,121,122], and water [121,123]. The SPME device consists of a syringe-like unit containing a fused silica fiber coated with a polymer or solid stationary phase which will hold the analytes from the sample by sorption. For air sampling, the fiber is exposed to the headspace of the extraction flask for a set period, after which it will be injected into the GC analytical instrument for thermal desorption and analysis. The selectivity of this technique depends heavily on the choice of the fiber coating, as factors such as polarity, stability, and thickness of the stationary phase will affect the results [124]. Fibers coated with polydimethylsiloxane (PDMS), carboxen (CAR), divinylbenzene (DVB) and polyacrylate, as well as combinations such as CAR/PDMS, PDMS/DVB and DVB/CAR/PDMS, are commercially available and often used for a variety of purposes [54,124]. These coatings allow for the analysis of a wide range of compounds, including aromatic hydrocarbons (benzene, toluene, xylenes), phenols, alcohols, ketones, and nitroaromatics. Derivatization of the SPME fiber may also enhance selectivity, sensitivity, and chromatographic separation [124,125].

Jordão et al. (2006) [126] studied VOC emissions from two different species of oak wood (*Quercus pyrenaica* and *Quercus petraea*) used in cooperage using SPME with a DVB/CAR/PDMS coated fiber and results showed that hexanal, furfural, α-pinene, d-limonene, and α-terpineol were detected by direct analysis from solid oak wood samples. Similarly, Bajer et al. (2019) [127] used a headspace-SPME with a DVB/CAR/PDMS coated fiber to characterize the volatile profile of stem wood in seven different species and identified 16 groups of VOCs. Although various studies showed that a fiber coated with DVB/CAR/PDMS achieved better results for VOC extraction in wood [47,53,126,127], PDMS-coated fibers have also been used for the same purpose [69,128]. In 2012, Liu et al. [69] extracted 14 different compounds (most of which were terpenes) from Dahurian larch *(Larix gmelinii)* particles using SPME with a PDMS-coated fiber, whereas Ewen et al. (2004) [128] performed SPME extractions on pine wood timber (*Pinus sylvestris*) and identified 12 VOCs by using a PDMS-coated fiber.

Recently, an innovative and versatile sample preparation technique which allows the extraction of volatile and semi-volatile compounds was used to extract volatile carbonyl compounds from different WBPs. Developed in 2010, gas-diffusion microextraction (GDME) is a simple, affordable, and non-exhaustive sample preparation technique that merges the concepts of membrane assisted gas-diffusion and microextraction, with the possibility of derivatization being part in the extraction process [129]. Similar to how the use of different fiber coatings affects selectivity in SPME, the use of different acceptor solutions in GDME allows for the analysis of targeted groups of compounds [130,131,132,133]. Through a GDME-HPLC-DAD-MS/MS methodology coupled with DNPH derivatization, Gonçalves et al. (2024) [98] identified 27 carbonyl compounds in commercial MDF samples, e.g., saturated aldehydes (C_1_–C_10_), unsaturated aldehydes (acrolein, crotonaldehyde), ketones (acetone, hydroxyheptanone, 2,3-pentanedione), dicarbonyl compounds (glyoxal, diacetyl), furfural, and benzaldehyde derivatives. Furthermore, a principal component analysis on volatile carbonyl compounds emitted from single-layered PBs produced with different particles (pine and recycled) and different resins (UF and UF fortified with melamine) showed that through the GDME technique it was possible to successfully distinguish PBs by their emissions, according to their differences in production [97].

Overall, alternative methodologies for VOCs determination offer faster and more affordable options compared to standard international methodologies. However, these are often performed under accelerated conditions, therefore not mimicking the indoor space at normal conditions. Among these alternative methodologies, classic extraction procedures are more complex and time-consuming, while miniaturized approaches like SPME and GDME provide simpler, faster, and more practical solutions for VOC analysis.

## 5. Comparison of Emission Results Found in the Literature

Due to the wide selection of analytical methods available for identification and/or determination of VOCs in wood and WBP, results are often reported in different units [70,74,134,135], as relative proportions [61,63,136,137], or simply identified without quantification [68,76,97,98]. Moreover, different testing conditions are commonly used. As a result, comparing VOC emission data is challenging, unless they share the same analytical method and somewhat similar testing conditions.

A more detailed compilation of VOCs found in different wood types and WBPs—including key information such as the analytical method employed for identification or determination, test conditions, VOC class, and results—is available in the Excel file provided as Supporting Information, which includes more than 2100 entries. A summarized version of this information, which compares the emissions of volatile compounds (e.g., terpenes, carbonyl, and other compounds) from different wood types and WBP based on the analysis method and experimental conditions, is presented in Table 9, Table 10 and Table S1.

Several recent studies highlight these comparative challenges and provide valuable emission data. In 2020, Czajka et al. [64] tested and compared VOC emissions from the heartwood and sapwood of Scots pine (*Pinus sylvestris*), European larch (*Larix decidua*), and Norway spruce (*Picea abies*), obtained at 3 and 28 days according to ISO 16000-6 and ISO 16000-9. After 3 days of testing, Scots pine exhibited the highest emissions for most compounds, namely camphene (heartwood, 198 µg·m^−3^), limonene (heartwood, 85 µg·m^−3^), α-pinene (heartwood, 11448 µg·m^−3^), Δ3-carene (heartwood: 951 µg·m^−3^; sapwood: 134 µg·m^−3^), hexanal (heartwood, 77 µg·m^−3^), octanal (heartwood, 23 µg·m^−3^), decanal (heartwood, 44 µg·m^−3^), benzaldehyde (heartwood, 16 µg·m^−3^), and acetophenone (heartwood, 106 µg·m^−3^). It was also the only sample to emit terpinolene (heartwood, 40 µg·m^−3^). European larch was the only sample reported to emit pentanal (sapwood, 29 µg·m^−3^), while Norway spruce showed the highest concentration of nonanal (heartwood, 61 µg·m^−3^).

Similarly, a comprehensive study by Schieweck, A. (2021) [91] using ISO 16000-3, VDI 4301-7 [140], and ISO 16000-9 evaluated VOC emissions from several different wood types and WBPs, such as laminated veneer lumber made from different wood types (beech and poplar) and adhesives (MUF, PF and polyvinyl acetate), plywood, PB, and OSB. Emissions were measured after a maximum testing period of three days. Among solid wood samples, oak presented lower emissions of most compounds, namely formaldehyde (7–29 µg·m^−3^), acetaldehyde (6–18 µg·m^−3^), propanal (<1 µg·m^−3^), acetone (<3–31 µg·m^−3^), formic acid (68–172 µg·m^−3^), and acetic acid (172–2876 µg·m^−3^). In contrast, solid pine wood showed some of the highest values for formaldehyde (28–82 µg·m^−3^), acetaldehyde (60–197 µg·m^−3^), propanal (4–13 µg·m^−3^), acetone (210–705 µg·m^−3^), formic acid (293–346 µg·m^−3^), and acetic acid (464–818 µg·m^−3^). Laminated veneer lumber samples had some of the lowest VOC emission values, as seen for formaldehyde (PF-beech: 2–6 µg·m^−3^; polyvinyl acetate-beech: 12–13 µg·m^−3^; MUF-beech: 16–23 µg·m^−3^), acetaldehyde (MUF-beech: 16–80 µg·m^−3^; MUF-poplar: 10–34 µg·m^−3^), acetone (PF-beech: 5–24 µg·m^−3^), propanal (<3 µg·m^−3^ for all samples), and formic acid (MUF-poplar: 19–44 µg·m^−3^). Commercial samples such as PB, plywood, and OSB had higher values and ranges for the majority of the determined compounds, for example, formaldehyde (PB: 39–243 µg·m^−3^; plywood: 15–201 µg·m^−3^), acetaldehyde (PB: 45–168 µg·m^−3^; plywood: 8–115 µg·m^−3^), acetone (PB: 136–424 µg·m^−3^; plywood: 8–127 µg·m^−3^; OSB: 95–517 µg·m^−3^), formic acid (plywood: 108–628 µg·m^−3^), and acetic acid (PB: 6–376 µg·m^−3^; plywood: 414–910 µg·m^−3^).

Baumann et al. (1999) [74] and Baumann et al. (2000) [94] used a different test chamber method (ASTM D 5116-90) to determine the area specific emission rates of PBs and MDFs produced with different wood-types for 4 days, which were mentioned in a previous section. Terpene emissions were mostly reported in WBPs containing pine wood (southern pine or other pine species), namely borneol (MDF-other pine: 7 µg·m^−2^·h^−1^; PB-other pine 12 µg·m^−2^·h^−1^), camphene (PB-southern pine: 3 µg·m^−2^·h^−1^), p-cymene (PB-southern pine: 6 µg·m^−2^·h^−1^) and α-pinene (PB-other pine: 23 µg·m^−2^·h^−1^). WBPs produced entirely by or containing hardwoods resulted in the lowest emissions for benzaldehyde (PB: 3 µg·m^−2^·h^−1^; MDF: 3 µg·m^−2^·h^−1^), pentanal (PB: 89 µg·m^−2^·h^−1^), heptanal (PB: 7 µg·m^−2^·h^−1^; MDF: 3 µg·m^−2^·h^−1^), nonanal (PB: 12 µg·m^−2^·h^−1^) and trans-2-octenal (MDF: 3 µg·m^−2^·h^−1^) when compared with the highest emission results obtained by MDFs produced with southern pine (benzaldehyde: 135 µg·m^−2^·h^−1^; pentanal: 228 µg·m^−2^·h^−1^; hexanal: 1781 µg·m^−2^·h^−1^; heptanal: 70 µg·m^−2^·h^−1^; nonanal: 96 µg·m^−2^·h^−1^; trans-2-octenal: 60 µg·m^−2^·h^−1^). However, when compared with PBs produced with southern pine, PBs manufactured with hardwoods exhibited higher results for hexanal (PB-hardwood: 1245 µg·m^−2^·h^−1^; PB-southern pine: 981 µg·m^−2^·h^−1^) and similar emissions of trans-2-octenal (PB-hardwood: 36 µg·m^−2^·h^−1^; PB-southern pine: 33 µg·m^−2^·h^−1^).

In summary, the literature consistently indicates that solid pine wood and WBPs produced with pine generally exhibit higher emissions of both terpenes and carbonyl compounds compared to other wood species. Specifically, MDFs and PBs made with southern pine species often report the highest area specific emission rates for many of the VOCs tested.

## 6. Final Remarks

Wood-based panels (WBPs) have become one of the most dynamic segments of the wood-processing industry, largely due to their role in reducing waste by reusing and recycling wood. However, wood and WBPs are associated with the emission of many volatile organic compounds (VOCs), which can negatively impact the indoor air quality of living and working spaces.

The volatile emission profile of virgin wood and WBP is affected by wood type and species, as well as by other factors such as age, storage, and/or drying conditions. Generally, softwoods exhibit higher emissions of volatile terpenes and lower emission of carbonyl compounds, carboxylic acids, and alcohols, when compared with hardwoods. In response to the occupational hazards from VOC exposure associated with these products, several methods have been developed and standardized for their determination. Most rely on the sorption of the volatiles, either through sorbent tubes (e.g., Tenax^®^), columns (solid-phase extraction, SPE) or fibers (solid-phase microextraction, SPME) followed by gas chromatography separation and mass spectrometric detection. Concurrently, new approaches (e.g., gas-diffusion microextraction, GDME) are being developed to offer more affordable and simpler alternatives for the targeted analysis of these compounds.

This review provides a multidisciplinary overview on WBPs, their production, classes, and adhesives. It also presents an updated summary of the current legislation and legal limits of emission, leading to the main topic of discussion focused on their VOC emission. Herein, the main classes of VOCs commonly associated with wood and WBPs are focused, along with their potential health hazards and the methods used for their determination.

## Figures and Tables

**Table 1:** List of applications, advantages, and disadvantages of the most used synthetic thermosetting resins. Adapted from [28,29,30,31,32].

Resin System	Applications	Advantages	Disadvantages
UF	PB MDF Plywood	Inexpensive; Fast curing; Adaptable to curing conditions; Water soluble; Hardness; Colorless when cured.	Low resistance to water; Emission of formaldehyde at slow rates.
MF & MUF	Exterior or semi-exterior plywood PB	Moisture tolerant; Durable; Less susceptible to release formaldehyde.	Expensive; Slow curing.
PF	Construction plywood OSB	Resistant to water; Durable; Fast curing; Low formaldehyde release.	Expensive; Dark colored when cured; Reduced mechanical properties.
pMDI	PB OSB MDF	Moisture tolerant; Fast curing; Durable; VOCs and formaldehyde-free.	Expensive; Adheres to metal; Potentially carcinogenic (when uncured).

**Table 2:** List of legal limits on VOC emissions from construction, decoration, and furniture products from different European countries, in accordance with the chamber method described in EN 16516 [42].

Country	Substance	Limits (mg·m^−3^)	References
Germany	Carcinogenic compounds (3 days)	0.01	[43,44]
	TVOCs (3 days)	10	
	Carcinogenic compounds (28 days)	0.001	
	TVOCs (28 days)	1	
Belgium	Formaldehyde (28 days)	0.1	[43,44]
	Acetaldehyde (28 days)	0.2	
	Toluene (28 days)	0.3	
	TVOCs (28 days)	1	
	Carcinogenic compounds (28 days)	0.001	
Italy	Formaldehyde (28 days)	0.06	[44]
	Acetaldehyde, Xylene (28 days)	0.3	
	Benzene (28 days)	0.001	
	Toluene (28 days)	0.45	
	TVOCs (28 days)	1.5	

**Table 3:** List of the French limits on VOC emissions from construction products, floorings, wall coverings, paints and varnishes, in accordance with the chamber method described in ISO 16000 series [43,44].

Substance	Limits (mg·m^−3^) After 28 Days of Emission Testing			
	Class C	Class B	Class A	Class A+
Formaldehyde	>0.12	0.06–0.12	0.01–0.06	<0.01
Acetaldehyde, Xylene	>0.4	0.3–0.4	0.2–0.3	<0.2
Toluene	>0.6	0.45–0.6	0.3–0.45	<0.3
TVOCs	>2	1.5–2	1–1.5	<1

**Table 4:** Volatile terpenes found in the most used wood types.

Wood	Volatile Terpenes Emitted *	References
Pine (*Pinus sylvestris*, *Pinus* spp.)	α-pinene, β-pinene, limonene, Δ3-carene, α-terpineol, camphene, β-phellandrene, terpinolene, verbenol, *p*-cymene, β-myrcene, and γ-terpinene	[50,51,61,62,63,64,65,66]
Spruce (*Picea abies*)	α-pinene, β-pinene, limonene, Δ3-carene, β-phellandrene, β-myrcene and camphene	[61,63,64,66]
Larch (*Larix decidua*, *Larix sibirica*, *Larix gmelinii)*	α-pinene, β-pinene, limonene, Δ3-carene, β-phellandrene, camphene, terpinolene, sabinene, tricyclene, α-phellandrene, linalool, τ-elemene, α-farnesene, β-caryophyllene, longifolene, β-cubinene, σ/τ-cadinene, α-caryophyllene, β-bourbonene and isolongifolene	[64,67,68,69]
Fir (*Abies alba*)	α-pinene, β-pinene, limonene, Δ3-carene, camphene, β-phellandrene and myrcene	[70]

* The order in which these compounds occur is not indicative of the reported emitted amounts.

**Table 5:** Volatile terpenes found in different wood-based panels.

Wood-Based Panel	Volatile Terpenes Emitted *	References
Particleboard (undisclosed pine species)	α-pinene, β-pinene, limonene, Δ3-carene, camphene, fenchone, fenchol, camphor, *p*-cymene, borneol	[74]
Particleboard (Douglas fir)	α-pinene, β-pinene, limonene, Δ3-carene, *p*-cymene, borneol	[74]
Particleboard (undisclosed hardwood species)	α-pinene, β-pinene, Δ3-carene	[74]
Particleboard (undisclosed wood species)	α-pinene, β-pinene, limonene, Δ3-carene, camphene, camphor, linalool, longifolene, *m*-cymene, *p*-cymene, *trans*-calamenene, α-muurolene, β-copaene, borneol, α-thujene	[75,76]
Particleboard and laminated particleboard (60% Dahurian larch, softwood, and hardwood mix)	α-pinene, limonene, Δ3-carene	[77]
MDF (undisclosed pine species)	limonene, Δ3-carene, borneol, *p*-cymene, fenchone, fenchol, camphor	[74]
MDF (undisclosed hardwood species)	limonene, *p*-cymene, borneol	[74]
MDF (undisclosed wood species)	α-pinene, β-pinene, limonene, Δ3-carene, camphene, camphor, linalool, longifolene, *p*-cymene, *trans*-calamenene, α-muurolene, β-copaene, borneol	[75]

* The order in which these compounds occur is not indicative of the reported emitted amounts.

**Table 6:** Volatile carbonyl compounds found in different common wood types.

Wood		Volatile Carbonyl Compounds Emitted *	References
Softwood	Pine (*Pinus sylvestris*, *Pinus* spp.)	formaldehyde, acetaldehyde, propanal, butanal, pentanal, hexanal, octanal, nonanal, decanal, oct-2-enal, furfural, benzaldehyde, acetone, 6-methyl-5-hepten-2-one, acetophenone, formic acid, acetic acid, octanoic acid, nonanoic acid	[48,64,65,87,90,91]
	Spruce (*Picea abies*)	formaldehyde, acetaldehyde, propanal, butanal, pentanal, hexanal, octanal, nonanal, furfural, benzaldehyde, acetone, acetophenone, formic acid, acetic acid	[48,64,90,91]
	Larch (*Larix decidua*, *Larix sibirica*, *Larix gmelinii)*	formaldehyde, acetaldehyde, octanal, nonanal, decanal, benzaldehyde, formic acid, acetic acid, acetone, acetophenone	[64,69,91]
Hardwood	Oak (*Quercus robur*)	formaldehyde, acetaldehyde, propanal, butanal, hexanal, furfural formic acid, acetic acid, hexanoic acid, methylacetate	[48,90,91,92]
	Beech (*Fagus sylvatica*)	formaldehyde, acetaldehyde, propanal, butanal, pentanal, hexanal, furfural, acetone, formic acid, acetic acid, hexanoic acid	[48,90,91,93]
	Ash (*Franxinus excelsior*)	formaldehyde, acetaldehyde, propanal, butanal, hexanal, acetic acid	[48]

* The order in which these compounds occur is not indicative of the reported emitted amounts.

**Table 7:** Volatile carbonyl compounds found in different wood-based panels.

Wood-Based Panel	Volatile Carbonyl Compounds Emitted *	References
Particleboard (undisclosed pine species)	formaldehyde, acetaldehyde, propanal, butanal, pentanal, hexanal, heptanal, octanal, nonanal, furfural, benzaldehyde, oct-2-enal	[94,97]
Particleboard (Douglas fir)	Pentanal, hexanal, octanal, nonanal, benzaldehyde, oct-2-enal	[94]
Particleboard (undisclosed hardwood species)	Pentanal, hexanal, heptanal, octanal, nonanal, benzaldehyde, oct-2-enal	[94]
Particleboard (undisclosed wood species)	formaldehyde, acetaldehyde, propanal, butanal, pentanal, hexanal, octanal, nonanal, decanal, furfural, benzaldehyde, propenal, oct-2-enal, acetone, butan-2-one	[75,90,94]
Particleboard and laminated particleboard (60% Dahurian larch, softwood, and hardwood mix)	Acetaldehyde, hexanal, heptanal, octanal, nonanal, decanal, undecanal, acetone, (5E)-6,10-Dimethylundeca-5,9-dien-2-one (geranylacetone)	[77]
MDF (undisclosed pine species)	Pentanal, hexanal, heptanal, octanal, nonanal, benzaldehyde, oct-2-enal	[94]
MDF (undisclosed hardwood species)	Pentanal, hexanal, octanal, nonanal, benzaldehyde, oct-2-enal	[94]
MDF (undisclosed wood species)	formaldehyde, acetaldehyde, propanal, butanal, pentanal, hexanal, heptanal, octanal, nonanal, decanal, benzaldehyde, oxaldehyde (glyoxal), 4-hydroxybenzaldehyde, prop-2-enal, but-2-enal, hept-2-enal, oct-2-enal, dec-2-enal, acetone, butan-2-one, 1-hydroxypropan-2-one (acetol), butane-2,3-dione, pentane-2,3-dione	[75,90,94,98]

* The order in which these compounds occur is not indicative of the reported emitted amounts.

**Table 8:** Other volatile compounds found in different common wood types.

Wood		Other Volatile Compounds Emitted *	References
Softwood	Pine (*Pinus sylvestris*, *Pinus* spp.)	ethanol, pentan-1-ol, tridecane, toluene, *o*/*m*/*p*-xylene	[64,65,87,91]
	Spruce (*Picea abies*)	ethanol, propan-2-ol, 2-ethylhexanol, *o*/*m*/*p*-xylene	[64,91]
	Larch (*Larix decidua*, *Larix sibirica*, *Larix gmelinii)*	ethanol, propan-2-ol, 2-ethylhexanol, 1-propene, octadecane, *o*/*m*/*p*-xylene	[64,69,91]
Hardwood	Oak (*Quercus robur*)	methanol, ethanol, propan-2-ol, 2-penthylfuran	[48,91]
	Beech (*Fagus sylvatica*)	methanol, ethanol, propan-2-ol, 2-penthylfuran	[48,91,92]
	Ash (*Franxinus excelsior*)	methanol, ethanol, 2-penthylfuran	[48,92]

* The order in which these compounds occur is not indicative of the reported emitted amounts.

**Table 9:** Summary of methods and measured values for terpenes found in wood and WBP.

Wood	Conditions	Compounds	Value	Compounds	Value	Unit	Method	References
**Douglas Fir**								
Particleboard	Conditioned (23 °C, 45% RH) 4 days of sampling period	α-Pinene	11	Limonene	9	µg·m^−2^·h^−1^	GC-MSD	[74]
		β-Pinene	1	Borneol	4			
		Δ3-Carene	2	*p*-Cymene	4			
		α-Pinene/Δ3-Carene	8	Borneol	4		ASTM D 5116-90 [138]	
		β-Pinene/Limonene	38	*p*-Cymene	8			
**Fir**								
Solid Wood	Untreated Age of wood: 1 year	α-Pinene	115 ± 1	Cymene	6.16 ± 0.05	mg·kg^−1^	Hexane extraction and GC-MS	[70]
		β-Pinene	20.99 ± 0.09	Borneol	3.34 ± 0.08			
		Limonene	6.2 ± 0.1	Fenchol	2.4 ± 0. 2			
		Camphene	12.3 ± 0.3	Thymol	4.3 ± 0.1			
		α-Phellandrene	1.65 ± 0.04	Myrtenal	6.1 ± 0.2			
		Verbenone	7.6 ± 0.1					
	Heat treatment (120 °C) Age of wood: 1 year	α-Pinene	0.175 ± 0.003	Cymene	0.0252 ± 0.0009			
		β-Pinene	0.067 ± 0.001	Borneol	0.0163 ± 0.0002			
		Limonene	0.0413 ± 0.0004	Fenchol	0.0077 ± 0.0001			
		Camphene	0.0059 ± 0.0002	Thymol	0.040 ± 0.002			
		α-Phellandrene	0.169 ± 0.003	Myrtenal	0.0459 ± 0.0009			
		Verbenone	0.455 ± 0.009					
	Untreated	α-Pinene	4.33 ± 0.03	Verbenone	0.80 ± 0.02			
	Age of wood: 146 years	β-Pinene	0.192 ± 0.003	Thymol	0.52 ± 0.01			
		Limonene	1.0672 ± 0.013	Myrtenal	1.2875 ± 0.004			
		Camphene	0.387 ± 0.004					
	Heat treatment (120 °C) Age of wood: 146 years	Limonene	0.337 ± 0.006	Borneol	0.6303 ± 0.006			
**Pine**								
Solid wood	Air-dried Conditioned (23 °C, 50% RH) 28 days of sampling period	α-Pinene	410	Camphene	9	µg·m^−2^·h^−1^	ISO 16000-6 and GC-MS	[66]
		β-Pinene	17	β-Phellandrene	11			
		Δ3-Carene	340	β-Myrsene	17			
		Limonene	13					
	Heat treatment (212 °C) Conditioned (23 °C, 50% RH) 28 days of sampling period	α-Pinene	2	Limonene/Δ3-Carene	3			
Heartwood/ Sapwood	Fresh wood Conditioned (23 °C, 45% RH) 28 days of sampling period	α-Pinene	459/294	Limonene	5/<1	µg·m^−3^	ISO 16000-6 and ISO 16000-9	[64]
		β-Pinene	13/15	Camphene	23/10			
		Δ3-Carene	108/40	Terpinolene	8/-			
	Air-dried Conditioned (20–25 °C, 50% RH) 3 h of sampling period	β-Pinene	7.95/2.82	Camphene	8.61/1.73	mg·m^−2^·h^−1^	ISO 16000-6, ISO 16000-10 [139] and GC-MS	[62]
		Limonene	5.26/1.22	γ-Terpinene	-/0.02			
	Heat treatment (200 °C) Conditioned (23 °C, 45% RH) 3 h of sampling period	β-Pinene	3.59/4.73	γ-Terpinene	0.17/0.06			
		Limonene	5.62/2.41	α-Phellandrene	0.125/0.05			
Particleboard	Conditioned (23 °C, 45% RH) 4 days of sampling period	α-Pinene	42	Camphene	3	µg·m^−2^·h^−1^	GC-MSD	[74]
		β-Pinene	72	Borneol	11			
		Δ3-Carene	78	*p*-Cymene	28			
		Limonene	50					
		α-Pinene	23	Camphene	2		ASTM D 5116-90	
		β-Pinene	26	Borneol	12			
		Δ3-Carene	48	*p*-Cymene	24			
		Limonene	31					
MDF	Conditioned (23 °C, 45% RH) 4 days of sampling period	Δ3-Carene	2	Borneol	7		ASTM D 5116-90	
		Limonene	2	*p*-Cymene	0.2			
**Spruce**								[66]
Solid wood	Air-dried Conditioned (23 °C, 50% RH) 28 days of sampling period	α-Pinene	54	Camphene/β-Myrsene	4	µg·m^−2^·h^−1^	ISO 16000-6 and GC-MS	
		β-Pinene	38	Limonene	53			
		Δ3-Carene	6	β-Fellandrene	5			
	Heat treatment (190 °C) Conditioned (23 °C, 50% RH) 28 days of sampling period	α-Pinene	3	Limonene	1			

**Table 10:** Summary of methods and measured values for carbonyl compounds found in wood and WBP.

Wood	Conditions	Compounds	Value	Compounds	Value	Unit	Method	References
**Aspen**								
Solid wood	Air-dried Conditioned (23 °C, 50% RH) 28 days of sampling period	Acetic acid	51	Propanoic acid	5	µg·m^−2^·h^−1^	ISO 16000-6 and GC-MS	[66]
		Hexanoic acid	9	Benzoic acid	1			
		Pentanal	29	Hexanal	180			
		Pent-2-enal	4					
	Heat treatment (190 °C)	Acetic acid	170	Propanoic acid	7			
	Conditioned (23 °C, 50% RH)	Benzoic acid	2	Furfural	37			
	28 days of sampling period	Pentanal/Hexanal	1	Octanal	1			
		Nonanal/Decanal	2					
**Beech**								
Solid wood (Veneer)	Conditioned (23 °C, 50% RH) 2.5 h of sampling period	Formic acid	252–366	Acetic acid	1104–1166	µg·m^−3^	VDI 4301-7 [140] and ISO 16000-9	[91]
		Formaldehyde	6–12	Propanal	<3		ISO 16000-3 and ISO 16000-9	
		Acetaldehyde	13–17	Acetone	4–98			
		Prop-2-enal	<1				ISO 16000-9 and according to [141]	
Solid wood (Slat)	Conditioned (23 °C, 50% RH) 2.5 h of sampling period	Formic acid	132–193	Acetic acid	1277–1720	µg·m^−3^	VDI 4301-7 and ISO 16000-9	
		Formaldehyde	<2	Propanal	7–13		ISO 16000-3 and ISO 16000-9	
		Acetaldehyde	16–48	Acetone	95–168			
		Prop-2-enal	<1				ISO 16000-9 and according to [141]	
Laminated veneer lumber	Conditioned (23 °C, 50% RH) 2.5 h of sampling period Resin: MUF	Formic acid	156–400	Acetic acid	1302–1409	µg·m^−3^	VDI 4301-7 and ISO 16000-9	
		Formaldehyde	16–23	Acetaldehyde	16–80		ISO 16000-3 and ISO 16000-9	
		Propanal	<3	Acetone	3–120			
		Prop-2-enal	<1				ISO 16000-9 and according to [141]	
	Conditioned (23 °C, 50% RH) 2.5 h of sampling period Resin: Phenol-Formaldehyde	Formic acid	217–276	Acetic acid	1268–1624	µg·m^−3^	VDI 4301-7 and ISO 16000-9	
		Formaldehyde	2–6	Acetaldehyde	36–161		ISO 16000-3 and ISO 16000-9	
		Propanal	<3	Acetone	5–24			
		Prop-2-enal	<1				ISO 16000-9 and according to [141]	
**Douglas fir**								
Solid wood	Conditioned (23 °C, 50% RH) 2.5 h of sampling period	Formic acid	28–106	Acetic acid	458–782	µg·m^−3^	VDI 4301-7 and ISO 16000-9	[91]
		Formaldehyde	11–23	Propanal	<3		ISO 16000-3 and ISO 16000-9	
		Acetaldehyde	20–70	Acetone	32–68			
		Prop-2-enal	1–4				ISO 16000-9 and according to [141]	
**Larch**								
Solid wood	Conditioned (23 °C, 50% RH) 2.5 h of sampling period	Formic acid	36–58	Acetic acid	286–352	µg·m^−3^	VDI 4301-7 and ISO 16000-9	[91]
		Formaldehyde	10–28	Propanal	<4		ISO 16000-3 and ISO 16000-9	
		Acetaldehyde	21–23	Acetone	9–27			
		Prop-2-enal	1–4				ISO 16000-9 and according to [141]	
**Oak**								
Solid wood	Conditioned (23 °C, 50% RH) 2.5 h of sampling period	Formic acid	68–172	Acetic acid	172–2876	µg·m^−3^	VDI 4301-7 and ISO 16000-9	[91]
		Formaldehyde	7–29	Propanal	<4–6		ISO 16000-3 and ISO 16000-9	
		Acetaldehyde	6–18	Acetone	<3–31			
		Prop-2-enal	<1				ISO 16000-9 and according to [141]	
Heartwood	Raw byproduct of wine barrels production (12% moisture content) Conditioned (23 °C, 50% RH) 18 h of sampling period	Acetic acid	(106 ± 15)·10^4^	Acetone	<0.4	µg·m^−2^·h^−1^	ISO 16000-9 and HPLC-DAD	[142]
		Formaldehyde	186.9 ± 9.0	Propenal	1080 ± 330			
		Acetaldehyde	313 ± 73	Furfural	(360 ± 50)·10^2^			
		Propanal	13.4 ± 1.1	Benzaldehyde	<1.5			
		Butanal	5.1 ± 4.0					
Binderless board	Heartwood Pressing conditions (170 °C, 30 MPa, 6 min) Conditioned (23 °C, 50% RH) 18 h of sampling period	Acetic acid	387 ± 68	Acetone	6.5 ± 1.2			
		Formaldehyde	60.5 ± 9.4	Propenal	187 ± 57			
		Acetaldehyde	782 ± 97	Furfural	337 ± 58			
		Propanal	11.0 ± 2.7	Benzaldehyde	<0.3			
		Butanal	13.8 ± 3.8					
**Pine**								
Solid wood	Air-dried Conditioned (23 °C, 50% RH) 28 days of sampling period	Acetic acid	3	1- Pentanol	12	µg·m^−2^·h^−1^	ISO 16000-6 and GC-MS	[66]
		Hexanal	34	Furfural	1			
	Heat treatment (212 °C) Conditioned (23 °C, 50% RH) 28 days of sampling period	Acetic acid	27	Benzoic acid	12			
		Pentanal	2	Benzaldehyde	6			
		Hexanal	11	Furfural	18			
		Nonanal	3					
Heartwood/ Sapwood	Fresh wood Conditioned (23 °C, 45% RH) 28 days of sampling period	Hexanal	4/152	Decanal	1/11	µg·m^−3^	ISO 16000-6 and ISO 16000-9	[64]
		Octanal	1/7	Benzaldehyde	<1/6			
		Nonanal	14/12	Acetophenone	1/11			
Particleboard	Conditioned (23 °C, 45% RH) 3 days of sampling period Average values	Pentanal	82	Nonanal	32	µg·m^−2^· h^−1^	ASTM D 5116-90	[94]
		Hexanal	851	trans-2-octenal	41			
		Heptanal	21	Benzaldehyde	55			
		Octanal	34					
MDF	Conditioned (23 °C, 45% RH) 3 days of sampling period	Pentanal	26	Nonanal	12			
		Hexanal	285	trans-2-octenal	4			
		Heptanal	7	Benzaldehyde	17			
		Octanal	9					
**Poplar**								
Laminated veneer lumber	Conditioned (23 °C, 50% RH) 2.5 h of sampling period Resin: MUF	Formic acid	19–44	Acetic acid	949–1486	µg·m^−3^	VDI 4301-7 and ISO 16000-9	[91]
		Formaldehyde	20–30	Acetaldehyde	10–34		ISO 16000-3 and ISO 16000-9	
		Propanal	<3	Acetone	3–120			
		Prop-2-enal	<1				ISO 16000-9 and according to [141]	
**Spruce**								
Solid wood	Air-dried Conditioned (23 °C, 50% RH) 28 days of sampling period	Acetic acid	12	Hexanal	8	µg·m^−2^·h^−1^	ISO 16000-6 and GC-MS	[66]
	Heat treatment (190 °C)	Acetic acid	28	Decanal	5			
	Conditioned (23 °C, 50% RH)	Hexanal	1	Furfural	23			
	28 days of sampling period	Nonanal	4					

## Data Availability

No new data were created or analyzed in this study. Data sharing is not applicable to this article.

## Supplementary Materials

The following supporting information can be downloaded at: https://www.mdpi.com/article/10.3390/molecules30153195/s1, File S1: Compilation of VOCs found in different wood types and WBP; Table S1: Summary of methods and measured values for other compounds found in wood and WBP.
