# Supplementary material for: Wood-Based Panels and Volatile Organic Compounds (VOCs): An Overview on Production, Emission Sources and Analysis
Source: Molecules. 2025 Jul 30;30(15):3195. doi: 10.3390/molecules30153195 (PMC12348141; doi:10.3390/molecules30153195)
Supplement: Supplementary file 1 [file molecules-30-03195-s001.zip › Table S1.pdf]

SUPPORT INFORMATION

Wood-Based Panels and Volatile Organic Compounds (VOCs): An Overview on Production, Emission Sources and Analysis

Fátima Daniela Gonçalves[1], Luísa Hora Carvalho[2,3,4], José António Rodrigues[1], Rui Miguel Ramos[1,5]*

[1]LAQV-REQUIMTE - Departamento de Química e Bioquímica, Faculdade de Ciências, Universidade do Porto, Rua do Campo Alegre, s/n, 4169-007 Porto, Portugal

[2]DEMad - Departamento de Engenharia de Madeiras, Instituto Politécnico de Viseu, Campus Politécnico de Repeses, 3504-510 Viseu, Portugal

[3]LEPABE – Faculty of Engineering, University of Porto, Rua Dr. Roberto Frias, s/n, 4200-465 Porto, Portugal

[4]ALiCE – Associate Laboratory in Chemical Engineering, Faculty of Engineering, University of Porto, Rua Dr. Roberto Frias, 4200-465 Porto, Portugal

[5]ARCP CoLAB – Rede de Competência em Polímeros, UPTEC Asprela II, Rua Júlio de Matos 828/882, 4200-355 Porto, Portugal

Table S1. Summary of methods and measured values for other compounds found in wood and WBP.

Wood	Conditions	Compounds	Value	Compounds	Value	Unit	Method	References
**Aspen**								
Solid wood	Air dried<br>Conditioned (23 ºC, 50% RH)<br>28 days of sampling period	1-Pentanol<br>1-Hexanol	18<br>8	1-Penten-3-ol<br>2-Ethyl-furane	10<br>5	µg m^{-2} h^{-1}	ISO 16000-6 and GC-MS	[1]
	Heat treatment (190 ºC)<br>Conditioned (23 ºC, 50% RH)<br>28 days of sampling period	Toluene	2	2-Ethylhexylether	2			
**Beech**								
Solid wood<br>(Veneer)	Conditioned (23 ºC, 50% RH)<br>2.5 hours of sampling period	Ethanol<br>Propan-2-ol<br>n-Pentane	8–49<br>5–25<br>< 2–2	Methyl acetate<br>2-Methylbutane	6<br>< 2–7	µg m^{-3}	ISO 16000-9 and according to [2]	[3]
Solid wood<br>(Slat)	Conditioned (23 ºC, 50% RH)<br>2.5 hours of sampling period	Ethanol<br>Propan-2-ol<br>n-Pentane	32–101<br>19–29<br>< 2	Methyl acetate<br>2-Methylbutane	8–11<br>< 2			
Laminated<br>veneer<br>lumber	Conditioned (23 ºC, 50% RH)<br>2.5 hours of sampling period<br>Resin: MUF	Ethanol<br>Propan-2-ol<br>n-Pentane	10–56<br>< 3–21<br>< 2	Methyl acetate<br>2-Methylbutane	9–13<br>< 2–6			
	Conditioned (23 ºC, 50% RH)<br>2.5 hours of sampling period<br>Resin: Phenol-Formaldehyde	Ethanol<br>Propan-2-ol<br>n-Pentane	14–81<br>3–31<br>< 2–2	Methyl acetate<br>2-Methylbutane	17–30<br>< 2–9			
**Douglas fir**								
Solid wood	Conditioned (23 ºC, 50% RH)<br>2.5 hours of sampling period	Ethanol<br>Propan-2-ol<br>n-Pentane	7–63<br>13–45<br>< 2–18	Methyl acetate<br>2-Methylbutane	18–35<br>5–282	µg m^{-3}	ISO 16000-9 and according to [2]	[3]
**Larch**								
Solid wood	Conditioned (23 ºC, 50% RH)<br>2.5 hours of sampling period	Ethanol<br>Propan-2-ol<br>n-Pentane	21–151<br>< 3–17<br>< 2-2	Methyl acetate<br>2-Methylbutane	< 3–5<br>2–144	µg m^{-3}	ISO 16000-9 and according to [2]	[3]
**Pine**								
Solid wood	Air dried<br>Conditioned (23 ºC, 50% RH)<br>28 days of sampling period	1-Methyl-2-iso-<br>-propylbenzene	19	Methyl(1-methylethenyl)-<br>-benzene	22	µg m^{-2} h^{-1}	ISO 16000-6 and GC-MS	[1]
	Heat treatment (212 ºC)<br>Conditioned (23 ºC, 50% RH)<br>28 days of sampling period	1-Metyhyl-2-iso-<br>-propylibenzene<br>Tetrahydrofurane	2<br>2	bis(2-Ethylhexyl)ether	3			
Heartwood/<br>Sapwood	Fresh wood<br>Conditioned (23 ºC, 45% RH)<br>28 days of sampling period	Toluene<br>*m*/*p*-Xylene<br>*o*-Xylene	7 / 6<br>1 / 12<br>< 1 / < 1	*n*-Undecane<br>*n*-Tetradecane	< 1 / 5<br>< 1 / < 1	µg m^{-3}	ISO 16000-6 and ISO 16000-9	[4]
	Air dried<br>Conditioned (20-25 ºC, 50% RH)<br>3 hours of sampling	1,2,3-Trimethylbenzene<br>2-Ethyltoluene	0.17 / –<br>0.09 / 0.06	Tetrachlorethylene	0.02 / 0.03	mg m^{-2} h^{-1}	ISO 16000-6, ISO 16000-10 and GC-MS	[5]
	Heat treatment (200 ºC)<br>Conditioned (23 ºC, 45% RH)<br>3 hours of sampling	Benzene<br>1,2,3-Trimethylbenzene<br>1,3,5-Trimethylbenzene<br>1-Methoxy-2-Propanol	– / 0.025<br>0.07 / 0.06<br>0.02 / 0.03<br>0.62 / –	2-Ethyltoluene<br>4-Ethyltoluene<br>Tetrachlorethylene	0.075 / 0.08<br>0.05 / 0.04<br>0.06 / 0.07			
**Spruce**								
Solid wood	Air dried<br>Conditioned (23 ºC, 50% RH)<br>28 days of sampling period	1-Methyl-4-<br>-(1-methylethyl)-benzene	6	1-Methyl-4-<br>-(1-methylethenyl)-benzene	5	µg m^{-2} h^{-1}	ISO 16000-6 and GC-MS	[1]
	Heat treatment (190 ºC)<br>Conditioned (23 ºC, 50% RH)<br>28 days of sampling period	bis(2-Ethylhexyl)ether	3					

References

[1] M. Hyttinen, M. Masalin-Weijo, P. Kalliokoski, P. Pasanen, Comparison of VOC emissions between air-dried and heat-treated Norway spruce (Picea abies), Scots pine (Pinus sylvesteris) and European aspen (Populus tremula) wood, Atmospheric Environment 44(38) (2010) 5028-5033.

[2] A. Schieweck, J. Gunschera, D. Varol, T. Salthammer, Analytical procedure for the determination of very volatile organic compounds (C3–C6) in indoor air, Analytical and Bioanalytical Chemistry 410(13) (2018) 3171-3183.

[3] A. Schieweck, Very volatile organic compounds (VVOC) as emissions from wooden materials and in indoor air of new prefabricated wooden houses, Building and Environment 190 (2021) 107537.

[4] M. Czajka, B. Fabisiak, E. Fabisiak, Emission of Volatile Organic Compounds from Heartwood and Sapwood of Selected Coniferous Species, Forests 11(1) (2020) 92.

[5] H. Sivrikaya, D. Tesařová, E. Jeřábková, A. Can, Color change and emission of volatile organic compounds from Scots pine exposed to heat and vacuum-heat treatment, Journal of Building Engineering 26 (2019) 100918.
